# Role of TG2-Mediated SERCA2 Serotonylation on Hypoxic Pulmonary Vein Remodeling

**DOI:** 10.3389/fphar.2019.01611

**Published:** 2020-02-11

**Authors:** Bo Liu, Dong Wang, Erfei Luo, Jiantong Hou, Yong Qiao, Gaoliang Yan, Qingjie Wang, Chengchun Tang

**Affiliations:** ^1^ Department of Cardiology, Zhongda Hospital, Southeast University, Nanjing, China; ^2^ Department of Cardiology, Changzhou No. 2 People’s Hospital, Nanjing Medical University, Changzhou, China

**Keywords:** serotonylation, TG2, SERCA2, pulmonary venous smooth muscle cell, store-operated calcium entry, [Ca2+]i

## Abstract

Sarco-endoplasmic reticulum Ca2+ ATPase (SERCA) pumps take up Ca2+ from the cytoplasm to maintain the balance of intracellular Ca2+. A decline in expression or activity of SERCA results in persistent store-operated calcium entry (SOCE). In cardiomyocytes as well as vascular smooth muscle cells (SMCs), SERCA2 acts as an important regulator of calcium cycling. The purpose of this study is to identify and better understand the role of transglutaminases2 (TG2) as a key factor involved in SERCA2 serotonination (s-SERCA2) and to elucidate the underlying mechanism of action. Human pulmonary venous smooth muscle cell in normal pulmonary lobe were isolated and cultured *in vitro*. Establishment of hypoxic pulmonary hypertension model in wild type and TG2 knockout mice. SERCA2 serotonylation was analyzed by co-(immunoprecipitation) IP when the TG2 gene silenced or overexpressed under normoxia and hypoxia *in vivo* and *in vitro*. Intracellular calcium ion was measured by using Fluo-4AM probe under normoxia and hypoxia. Real-time (RT)-PCR and Western blot analyzed expression of TG2, TRPC1, and TRPC6 under normoxia and hypoxia. Bioactivity of cells were analyzed by using Cell Counting Kit (CCK)-8, flow cytometry, wound healing, RT-PCR, and Western blot under PST-2744 and cyclopiazonic acid. We confirmed that 1) hypoxia enhanced the expression and activity of TG2, and 2) hypoxia increased the basal intracellular Ca2+ concentration ([Ca2+]i) and SOCE through activating TRPC6 on human pulmonary vein smooth muscle cells (hPVSMC). Then, we investigated the effects of overexpression and downregulation of the TG2 gene on the activity of SERCA2, s-SERCA2, basal [Ca2+]i, and SOCE under normoxia and hypoxia *in vitro*, and investigated the activity of SERCA2 and s-SERCA2 *in vivo*, respectively. We confirmed that SERCA2 serotonylation inhibited the activity of SERCA2 and increased the Ca2+ influx, and that hypoxia induced TG2-mediated SERCA2 serotonylation both *in vivo* and *in vitro*. Furthermore, we investigated the effect of TG2 activity on the biological behavior of hPVSMC by using an inhibitor and agonist of SERCA2, respectively. Finally, we confirmed that chronic hypoxia cannot increase vessel wall thickness, the right ventricular systolic pressure (RVSP), and right ventricular hypertrophy index (RVHI) of vascular smooth muscle-specific Tgm2−/− mice. These results indicated that hypoxia promoted TG2-mediated SERCA2 serotonylation, thereby leading to inhibition of SERCA2 activity, which further increased the calcium influx through the TRPC6 channel. Furthermore, tissue-specific conditional TG2 knockout mice prevents the development of pulmonary hypertension caused by hypoxia. In summary, we uncovered a new target (TG2) for treatment of chronic hypoxic pulmonary hypertension (CHPH).

## Introduction

Pulmonary arterial hypertension (PAH) is a condition marked by high blood pressure in the lungs and vascular remodeling, which can lead to right heart failure (RHF), and even death. Vascular remodeling in the lungs is characterized by the excessive proliferation, movement, and blockage of apoptosis of pulmonary vascular smooth muscle cells (Dai et al., 2018). In previous studies, many researchers paid close attention to the role of pulmonary arteries on PAH, however more recently more and more studies have revealed that pulmonary veins (PVs) also play an important role on PAH (Jin et al.2013; Zhao et al.,1993; Kulik, 2014). Chronic hypoxic exposure, vasoconstriction, and structural alterations occur not only in pulmonary arteries (PAs) but also in PVs, each contributing significantly to total pulmonary vascular resistance (Raj and Chen, 1986; Zhao et al., 1993; Raj et al., 1990, 1992; Gao and Raj, 2005; Peng et al., 2010). In addition, intrapulmonary arteries and veins contributed to the increase in pulmonary vascular resistance during hypoxia (Aguero et al., 2016; Nelson et al., 2016; Sahoo et al., 2015). The abnormal proliferation and migration of pulmonary vein smooth muscle cells (PVSMCs) formed the pathological basis of pulmonary vein remodeling. In our previous study, we indicated that transient receptor potential cation 6 (TRPC6), not TRPC1, was functionally upregulated in rat PVs and PVSMCs in response to chronic hypoxia (CH). There is evidence to suggest that TRPC6 channels are activated, in the setting of hypoxia, by both stores operated and receptor operated mechanisms, however the relative contribution of each to TRPC6 activity remains contentious. It was widely believed that ROCE produced a large amount of calcium influx in a short time, causing acute pulmonary vessels vasoconstriction, and SOCE produced a small and persistent calcium influx which played an important role on chronic pulmonary vascular remodeling (Yuan et al., 2009; Xu et al., 2014; Peng et al., 2015). Hypoxia increased the proliferation and migration of PVSMCs, which was attenuated by siTRPC6 (Xu et al., 2014). After the endoplasmic reticulum (ER) has released Ca2+, the enhanced intracellular Ca2+ concentration ([Ca2+]i) is maintained by Ca2+ from the store-operated Ca2+ entry (SOCE). SOCE regulates adding Ca2+ back into the ER, which prevents Ca2+ from entering and enables restorative resting of Ca2+. This exhibits a mediating effect on [Ca2+]i, which facilitates the resting [Ca2+]i in the nanomolar range by way of sarco(endo)plasmic reticulum calcium ATPase (SERCA). SERCA activity increases upon the release of ER/sarcoplasmatic reticulum (SR) Ca2+, which allows for rapid re-uptake of cytosolic Ca2+ (Luo et al., 1994). Subtype SERCA2 was a key modulator of calcium cycling in both cardiomyocytes and vascular SMCs, and the pulmonary arterial SERCA2 expression is down-regulated in a rat monocrotaline model of PH as well as in humans with PAH (Sahoo et al., 2015). 5-Hydroxytryptamine (5-HT and serotonin) is a well-known small protein that plays a central role in the pathogenesis of PAH and vascular remodeling (Lee et al., 1991; Lee et al., 1998; Lee et al., 1999; Liu et al., 2011; Thomas et al., 2013; Penumatsa et al., 2014a). Previous research has found that the enzyme tissue transglutaminase 2 (TG2) regulates the cross-linking of proteins with 5-HT. This is a post-translational process of monoaminylation, which is known as “serotonylation” TG2 activity and is active in both smooth muscle proliferation and contraction produced by 5-HT (Penumatsa et al., 2014b; He et al., 2016; Yu et al., 2016; Chen et al., 2018; Liu et al., 2018). Moreover, in our previous study, we used tandem mass spectrometry and immunoprecipitation of cardiomyocytes to confirm that SERCA2 was another target protein for 5-HT, and was named SERCA2 serotonylation (Wang et al., 2016). We speculated that CH inhibits the activity of SERCA2 by serotonylation, thereby activating TRPC6-mediated SOCE to increase the intracellular calcium concentration and promote cell proliferation and migration.

## Materials and Methods

### Reagents

Cyclopiazonic acid (CPA) and PST-2744 (SERCA2 agonists, Sigma-Aldrich, St. Louis, MO, USA), 5-BP, and Fluo-4 AM probes were from Life Technologies (Thermo Fisher Scientific, Waltham, MA, USA), culture–insert two well (ibidi GmbH, Germany).

### Small Interfering Ribonucleic Acid and Recombinant Adenovirus

hPVSMCs at 70–80% confluence was transfected with siRNA and recombinant adenovirus. Takes 2.5 μl (six-well) Lipofectamine^®^ RNAiMAX Reagent (Thermo Fisher Scientific) and 5 μl (six-well) siRNA into two centrifuge tubes with 250 μl serum-free and antibiotic-free DEME/F-12 medium separately, and blend by gently shaking. Then, add diluted siRNA to diluted Lipofectamine^®^ RNAiMAX Reagent, and incubate for 5 min at room temperature. Add siRNA-lipid complex to hPVSMCs (six-well) and then add 500 μl medium to six well for 1 ml. Replace fresh medium after 8 h and continue to culture hPVSMCs for 48 h.

Recombinant adenovirus (1×1,010 TU/ml) was diluted 1,000 times in medium. Add the diluted Recombinant Adenovirus to a six-well plate and add 1 ml to each well. Replace fresh medium after 12 h and continue to culture hPVSMCs for 72 h. hPVSMCs were treated with hypoxia after completing stable transfection. Small-interfering RNA (siRNA) molecules targeting human TRPC1, TRPC6, and TG2 messenger RNAs (mRNAs) were purchased from GenePharma biotech company, China. For TRPC1, TRPC6, and TG2 overexpression, adenoviral vectors were purchased from GenePharma biotech company, China. TRPC1 siRNA: sense strand: GGAUGUGCGGGAGGUGAAGTT; antisense strand: CUUCACCUCCCGCACAUCCTT; TRPC6 siRNA: sense strand: GCCACUCACUCAACGUUAATT; antisense strand: UUAACGUUGAGUGAGUGGCTT. TG2 siRNA: sense strand: GCCUGAUCCUUCUAGAUGUTT; antisense strand: ACAUCUAGAAGGAUCAGGCTT. The efficiency of the TG2 siRNA and adenovirus were about 68 and 230% mRNA, 15 and 170% protein. The efficiency of the TRPC1 siRNA and adenovirus were about 67 and 263% mRNA, 20 and 160% protein. The efficiency of the TRPC6 siRNA and adenovirus were about 71 and 233% mRNA, 17 and 211% protein. For specific analysis, please referred to **Supplementary Materials**.

### Vascular Smooth Muscle-Specific Tg2 Knockout Mice

Vascular smooth muscle-specific Tgm2 knockout mice were generated using standard Cre-LoxP-based gene targeting strategies. The final targeting vector for Tgm2 conditional knockout was constructed and subsequently delivered to vascular smooth cells. Male adult Tgm2 conditional knockout mice and female adult Tg(Tagln-cre)1Her/J were purchased from The Jackson Laboratory (USA) on a C57BL/6J background. These Tgm2t/t floxed mice possessed loxP sites flanking exons 6–8 of the transglutaminase 2, C polypeptide (Tgm2) gene. Under the control of mouse transgelin (smooth muscle protein 22-alpha), the transgenic mice expressed Cre recombinase. Cre-mediated recombination, when crossed with a strain possessing the appropriate loxP site-flanked sequence, will delete the flanked sequence in vascular smooth muscle cells. Mice were housed in groups of four in standard polypropylene cages in 12-h light-dark cycle approved by the Nanjing Medical University animal welfare guidelines (Nanjing, China). Genotyping was performed *via* PCR using DNA extracted from tail clippings with a Direct PCR (Tail) Kit (ViaGen Biotech Inc, USA). In brief, 8 to 10-week old male mice were used for *in vivo* experiments. Mice were assigned to experimental groups based on their genotype and SM22α-Cre+/− status. Selection of animal for CH treatment was performed randomly and in a blinded manner.

### Cell Isolation

Human pulmonary vein smooth muscle cells (hPVSMCs) were aseptically isolated from the intrapulmonary vein (fourth level) from surgical pulmonary lobectomy at room temperature. After removing adhering fat, connective tissue, and endothelial cells, the dissected media of the PVs was cut into small pieces (1–2 mm^2^) and covered by autoclaved glass coverslips in cell culture dishes. Next, we cultured fourth level hPVSMCs in DMEM/F-12 (HyClone, USA) which was supplemented with 10% fetal bovine serum (FBS) (Biological Industries, Israel), and 1% Penicillin-Streptomycin liquid (Gibco, USA) at 37°C and 5% CO2. PVSMCs were identified by positive immunostaining with anti-α-SMA monoclonal antibody (Abcam, UK). Cells at passages 2–3 were used in experiments, and each experiment was repeated at least three times with different preparations of cells. While the cells cultured in a tri-gas incubator, we performed hypoxia preconditioning (Forma Series II 3131 Water Jacket CO2 Incubator, Thermo Scientific, USA) for 12 or 24 h with oxygen concentration in 1% (Weisel, et al., 2014).

### Pulmonary Arterial Hypertension Model

*In vivo*, 8-week male wild-type (WT) mice (n = 6) and TG2 knockout (Tgm2−/−) mice (n = 6) were exposed to hypoxia (10% O2) in a normobaric chamber for 6 weeks, which represented the hypoxia group. Another set of identical WT mice (n = 6) and Tgm2−/− mice (n = 6) were kept under normal conditions for 6 weeks, and represented the normoxia groups. The chamber (AiPu XBS-02B, China) had an external oxygen controller, sensing the ambient oxygen concentration and replacing it with nitrogen when necessary. The chamber was partially ventilated. At the end of the treatment period, hemodynamic indexes were determined, and lung tissue of mice were collected (Chen et al., 2018; Liu et al., 2018).

### Hemodynamic and Morphometric Measurements

Mice were anesthetized with 1% pentobarbital sodium (80 mg/kg ip). Then the RSVP was measured by closed-chest insertion into the right ventricle (RV), in order to measure the mean pulmonary arterial pressure of spontaneously breathing, anesthetized animals using a xiphocostal approach. This entailed using a 22-gauge needle that was connected to a pressure transducer, both of which were controlled by the PowerLab system (ADInstruments, Australia). In order to specify the position of the needle, we used the wave form and our data were recorded using the Chart program (part of the PowerLab system). We then extracted the heart and lungs from the mice and separately weighed the right ventricle and the left ventricle plus interventricular septum (LV+S). This allowed us to examine the extent of RV hypertrophy. We calculated the right ventricular hypertrophy index (RVHI) according to the following formula: RVHI = [RV/(LV+S)]. Lung sections from inferior lobe of right lung were prepared and processed using hematoxylin-eosin (HE) staining using a standard protocol. Tissue sections were observed under a light microscope. At 400× magnification small pulmonary vessels of at least three animals per group ranging from 50 to 100 μm in internal diameter were assessed. The percentage medial layer thickness [MT% = 100 x (medial layer thickness)/(vessel semidiameter)] and area [MA% = 100 (cross-sectional medial layer area)/(total cross-sectional vessel area)] of peripheral pulmonary arteries were analyzed using a blind-method image-processing program (Image-Pro Plus, Version 6.0) (He et al., 2016; Chen et al., 2017).

### Co-Immunoprecipitation Assay

To determine the interaction between SERCA2 and 5-HT *in vivo* and *in vitro*, hPVSMCs were lysed in an immunoprecipitation (IP) lysis buffer (KeyGen BioTech, China) after incubation with 5-HT (1 mM) and Ca2+(6.7 mM). Cell lysates were cleared by centrifugation at 10,000 rpm for 5 min at 4°C. Briefly, distal (≥2th generations) PV relative to atrium were dissected from lungs of male mice after finishing hypoxia. The thin layer of adventitia was carefully stripped off with fine forceps, and the endothelium was wiped off using a cotton swab. Total protein was extracted using an immunoprecipitation (IP) lysis buffer (KeyGen BioTech, China) for distal PV tissue. The protein content was measured using a bicinchoninic acid assay (BCA) protein assay (KeyGen BioTech, China). In brief, magnetic beads were resuspended in the vial. And 50 µl Protein A/G magnetic beads were added to a 1.5 ml tube, and 150 µl binding buffer (50 mM Tris, 150 mM NaCl, 0.1% Triton X-100, pH 7.5) was added into the tube twice to perform magnetic separation. Next, 200 µl of 30 μg/ml anti-SERCA2 mouse monoclonal antibody (Abcam, UK) was added to pretreat the magnetic beads. The tube was rotated for 1 h at room temperature or 4 h at 4°C, then resuspended thoroughly by pipetting up and down. Magnetic separation was performed and the supernatant was discarded twice. Tubes were removed from the magnetic separator and the sample containing the antigen (Ag) (200 µl) was added and gently pipetted to resuspend the Protein A/G magnetic beads-Ab complex. The mixture was incubated overnight at 4°C under rotation to allow Ag to bind to the Protein A/G magnetic Bbads-Ab complex. Next, the magnetic beads-Ab-Ag complex was washed three times using 300 µl binding buffer per wash. Magnetic separation was performed between each wash, supernatant was removed and resuspended by gentle pipetting. The Protein A/G magnetic beads-Ab-Ag complex was resuspended in 150 µl binding buffer and the bead suspension were transferred to a clean tube to avoid co-elution of the proteins bound to the tube wall. Then, the supernatant was discarded and 30 μl of 1× sodium dodecyl sulfate polyacrylamide gel electrophoresis (SDS-PAGE) loading buffer was added, mixed well, and heated at 95°C for 5 min. The supernatant was collected for SDS-PAGE detection by magnetic separation, then 5-HT and SERCA2 expression were evaluated in the final supernatant using Western blot analysis using an anti-serotonin antibody (Abcam, UK) and anti-SERCA2 rabbit polyclonal antibody (Abcam, UK).

### SERCA2 Activity Assay

SERCA2 is a member of the ATPase family, which can decompose ATP into ADP and inorganic phosphorus. Analysis of the amount of inorganic phosphorus determined the level of ATPase activity by using ultramicro-Ca2+-ATPase detection kit (Jiancheng Bioengineering Institute, China). The treated hPVSMCs were digested, centrifuged, and the supernatant was removed, leaving layers of cells, and 200 μl of ultrapure water was added to each tube to prepare a 107/ml cell suspension, which was disrupted by an ultrasonic pulverizer. The prepared cell suspension did not centrifuge and the total protein concentration was measured by the BCA method. *In vivo*, pulmonary veins were isolated from WT and Tgm2−/− mice under hypoxia and normoxia. In brief, 9 vol of ultrapure water were added to the veins, and veins were mechanically disrupted in an ice bath, and centrifuged at 2,500 rpm for 10 min. Next, the supernatant was collected, diluted 10-fold, and the total protein concentration was measured by the BCA method. Enzymatic reactions and phosphorus determination were carried out according to the kit instructions. An UV spectrophotometer (Mapada UV-3100PC, China) was used to measure the absorbance of samples at a wavelength of 636 nm and 1 cm optical path after ultrapure water zero setting. The optical density (OD) value was used in the following formula to calculate the activity of ATPase. Activity of ATPase (U/mgprot) = (the OD value of the sample − the OD value of the control)/(OD value of standard-OD value of blank)×0.02 μmol/l×6×2.8÷total protein concentration of sample (mg protein/ml) (Guo et al., 2017).

### TG2 Activity Assay

For measurement of TG2 activity, 5-BP (400 μM, Thermo Scientific, USA) activity was visualized with fluorochrome-labeled HRP-added streptavidin. In brief, hPVSMCs were grown at a rate of 80% on glass coverslips (CitoGlas, China). hPVSMCs were exposed to 5-BP for an h prior to the exposure to hypoxia/normoxia, after 24 h of free-serum starvation. As a negative control, the incubation of 5-BP was omitted. Following the hypoxia/normoxia treatment, cells were fixed with 4% formaldehyde (Biosharp, China) for 20 min at room temperature. Cells were treated with 0.1% Triton X-100 for 30 min after complete removal of paraformaldehyde. Then, cells were blocked with 5% BSA in 0.1% Triton X-100 for 1 h at 4°C. Then, cells were incubated with 2 μg/L Streptavidin Alexa Fluor 488 Streptavidin HRP (YeaSen Bio, China) overnight at 4°C. Finally, cells were treated with 4′,6-diamidino-2-phenylindole (DAPI) (Leagene Biotechnology, China) at 37°C for 5 min, and was then sealed with nail polish. We examined the stained sections under a light microscope (Nikon Eclipse TE2000-S, Japan). The green fluorescence intensity per cell was calculated using ImageJ software to quantitatively analyze the activity of TG2 (Occhiogrosso et al., 2012).

### Measurements of [Ca2+]i and Store-Operated Calcium Entry

In order to quantify the [Ca2+]i in groups of hPVSMCs using an Infinite M200 PRO plate-reader (Tecan, Switzerland), the hPVSMCs were seeded into 96-well plates, with each well containing 104 cells. After 2 days, the medium was replaced with DMEM/F12 (with no serum), and the cells were used after incubation for another 24 h. Cells were washed with HEPES [4-(2-hydroxyethyl)-1-piperazineethanesulfonic acid]-buffered saline (HBS: 135 mM NaCl, 5.9 mM KCl, 1.2 mM MgCl2, 1.5 mM CaCl2, 11.6 mM HEPES, 11.5 mM D-glucose, pH 7.3), and loaded with ﬂuo-4 by incubation with Fluo-4-AM (2 μM) in HBS (100 μl per well). After 30 min at 37°C, HBS replaced the medium at a rate of 100 μl per well, and the fluorescence from Fluo-4AM (excitation 485 nm, emission 525 nM) was recorded at 5-min intervals for 30 cycles using Tecan i-control. For the measurement of SOCE, hPVSMCs were treated as in the following four steps. Step one, hPVSMCs were treated with HBS solution for 5 min. Step two, hPVSMCs were treated with free-Ca2+ HBS solution included 5 μM nifedipine (Aladdin Industrial Corporation, China) and 10 μM CPA (Sigma-Aldrich, St. Louis, MO, USA). Step three, hPVSMCs were treated with free-Ca2+ HBS solution included 5 μM nifedipine and 10 μM CPA for 10 min. Step four, hPVSMCs were treated with HBS solution for 10 min. Fluorescence was only recorded in steps 2 and 3. The change in intracellular Ca2+ concentration was represented by the change of fluorescence intensity. The ratio of fluorescence intensity (F/F0) was used to compare intracellular Ca2+ concentration under different treatments (F: the average fluorescence intensity under different treatments, F0: the initial fluorescence intensity) (Dale et al., 2018; Zhu et al., 2018).

### Real-Time Polymerase Chain Reaction

Total RNA from hPVSMCs with different treatments was isolated by using the RNAprep pure cell Kit (TianGen BioTech, China) according to the manufacturer’s protocol. The RNA concentrations were determined using a NanoDrop ND-1000 spectrophotometer (Thermo Scientific). Equal amounts of total RNA were reversed transcribed using the FastQuant RT Kit (with gDNase) (TianGen BioTech, China). RT-PCR was performed with the SuperReal PreMix Plus (SYBR Green) (TianGen BioTech, China) and Prism 7500 SDS (Applied Biosystems; Thermo Fisher Scientific, USA). The primers for TRPC1 were: h-TRPC1-s ATGTGCTTGGGAGAA ATGCTG, h-TRPC1-a TCTTGATGATCGTTTTGGTCG. The primers for TRPC6 were: h-TRPC6-sACTCCTTCCTAATGAAACCAGCAC, h-TRPC6-a CAGATTTCTTTACATTCAGCCCATA. The primers for h-β-actin were: h-ACTIN-s CACCCAGCACAATGAAGATCA AGAT, h-β-actin-a CCAGTTTTTAAATCCTGAGTCAAGC. The primers for TG2 were: h-TG2-s TATGGCCAGTGCTGGGTCTTCGCC, h-TG2-a GGCTCCAGGGTTAGGTTGAGCAGG. The relative gene expression values were calculated using the ΔΔCt method (ΔΔCt = ΔΔCt treated − ΔΔCt untreated control) and the equation y = 2 − ΔΔCt and β-actin served as the control.

### Lung Tissue Immunohistochemistry

Lung tissues were fixed in 4% paraformaldehyde, processed, and embedded in paraffin. We carefully examined the cores and inserted them into new paraffin blocks, using Tissue Arrayer Minicore (Alphelus, Plaisir, France). We deparaffinized the sections with a thickness of 5 μm and washed them with 100% ethanol, 90% ethanol, 70% ethanol, and then distilled water. These sections were then prepared for antigen retrieval in a citrate buffer with a pH of 6.0 by heating them in a microwave for 5-min cycles. The sections were then incubated overnight at a temperature of 4°C with a 1:200 diluted Anti-TG2 antibody (Abcam, USA), after which we incubated them with biotin-labeled Rabbit Anti-Mouse IgG H&L preabsorbed (Abcam, USA) for immunostaining (Chihong, et al., 2017).

### Western Blot Analysis

Protein samples were prepared in a similar way as was described for co-IP analysis. In brief, 30 μg of protein was loaded per lane, with a buffer of 8% SDS-PAGE gel subsequently transferred to polyvinylidene fluoride or polyvinylidene difluoride (PVDF) membranes. Following the transfer, the membranes were incubated overnight at 4°C with anti-TG2 monoclonal antibody (1:1,000, Abcam, UK), anti-osteopontin polyclonal antibody (1:1,000, Abcam, UK), anti-SM22α polyclonal antibody (1:1,000, Abcam, UK), anti-β-actin monoclonal antibody (1:1,000, Abcam, UK), and anti-calponin polyclonal antibody (1:1,000, Abcam, UK). Then, membranes were washed and incubated with an HRP-conjugated secondary antibody and developed using a ECL Substrate Kit (Invent Biotechnology, USA). The subsequent processes were performed according to the manufacturer’s instructions. The image was taken by a Tanon-4600 Chemiluminescent Imaging System (Tanon, China) for 1–3 min. Specific bands were analyzed according to apparent molecular sizes.

### Cell Proliferation Assay and Apoptosis Detection

FBS-free medium starvation for 24 h, hPVSMCs were treated with 10 μM cyclopiazonic acid (CPA) (SERCA2 inhibitor, Sigma-Aldrich, St. Louis, MO, USA) and 5 μM PST-2744 (SERCA2 agonists, Sigma-Aldrich, St. Louis, MO, USA) for 24 and 48 h, respectively. The proliferation of cells was examined using a Cell Counting Kit-8 assay (Dojindo, Japan). Cells were seeded at 5,000 cells/well into 96-well plates with 100 μl culture medium. Then, 10 μl of CCK-8 solution was added to the cells at specific time points and cells were incubated for 3 h at 37°C. Then, the absorbance at 450 nm was measured using a Microplate Reader (Bio-Rad, USA). Cells were seeded at 5×104 cells/well into six-well plates in 2,000 μl culture medium. Apoptosis was examined by flow cytometric analysis. An Annexin V-EGFP/PI double stain assay (KeyGen BioTech, China) was performed following the manufacturer’s protocol.

### Wound Scratch Assays

A total of 5 × 105 cells/ml hPVSMCs suspension was prepared as usual. Then, 70 µl of the suspension was placed into a culture–insert two well (ibidi GmbH, Germany). Shaking is avoided as this will result in an inhomogeneous cell distribution. Cells were incubated at 37°C and 5% CO2. After appropriate cell adherence (24 h), the culture–insert two well was gently removed by using sterile tweezers, and the outer area was filled with cell culture medium. In brief, hPVSMCs were seeded on two-wells silicone inserts with a defined cell-free gap culture dish, incubate at 37°C and 5% CO2 as usual, after appropriate cell attachment (24 h) gently remove the Culture-Insert 2 Well by using sterile tweezers. Grab a corner of the Culture-Insert 2 Well. Fill the used well or dish with cell free medium. The scratch healing area of cells was detected after 12 and 24 h and imaged under a microscope (Olympus, Japan).

## Statistical Analysis

Data are expressed as the mean ± SE; for at least three independent replicates (n ≥ 3). Data analyses were performed using either the Turkey and Dunnett test or the Student’s t test. A p value of less than 0.05 was considered statistically significant. Statistical analysis of the data was employed by one-way analysis of variance (ANOVA) using a post-test depending on the requirement.

## Results

### Effects on TG2 Expression and Activity From Hypoxia

**Figure 1:**
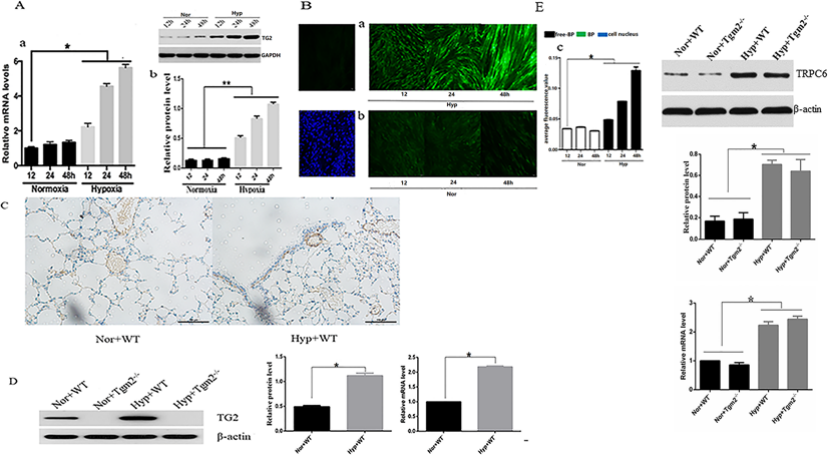
Effects of hypoxia on the expression and activity of TG2 *in vivo* and *in vitro*. All values are presented as the mean ± S.E.M. Nor, normoxia; Hyp, hypoxia; WT, wild type; Tgm2−/−. **(A**-a**)** The level of TG2 messenger RNA (mRNA) (n = 3, *p < 0.05 compared with the normoxic group). **(A**-b**)** TG2 protein band and the level of TG2 protein (n = 3, *p < 0.05 compared with the normoxic group, **p < 0.05 compared with normoxia). **(B)** Cells were exposed to 5-BP for 1 h prior to exposure to hypoxia/normoxia after 24 h of free-serum starvation. As a negative control, 5-BP incubation was omitted. Cells were then exposed to hypoxia or normoxia for 12, 24, and 48 h, and stained with streptavidin-conjugated BP incorporation, and 4′,6-diamidino-2-phenylindole (DAPI). The image shows the overlay of TG2 and 5-BP staining, which indicates the co-localization. **(B**-a**)** The green fluorescence was stronger after treatment with hypoxia and time-dependent. **(B**-b**)** Green fluorescence was weak and no marked differences in the normoxic group were observed. **(B**-c**)** The figure shows the relative fluorescence intensity (n = 3, *p < 0.05 as compared to the normoxic group). **(C)** TG2 location on the pulmonary vessels by using immunohistochemistry. **(D)** The level of TG2 protein and mRNA expression on the pulmonary veins under normoxia and hypoxia (n = 6, *p < 0.05 compared with the normoxic group). **(E)** The level of TRPC6 protein and mRNA expression on the pulmonary veins under normoxia and hypoxia (n = 6, *p < 0.05 compared with the normoxic group).

As shown in **Figure 1A**, no differences in expression of TG2 protein and mRNA were observed at various time points under normoxia. Compared with the normoxia group, expression of TG2 mRNA and protein significantly increased after hypoxia treatment for 12, 24, and 48 h and was time-dependent. As shown in **Figure 1B**, cells were stained with Alexa Fluor 488 Streptavidin-conjugated horseradish peroxidase (HRP) to detect TG2 localization. No differences in cytosolic fluorescence was observed at various time points under normoxia (**Figure 1B-b**). The streptavidin staining for (biotinamido)pentylamine (BP) incorporation showed a time dependent increased cytosolic fluorescence under hypoxia compared to the normoxia group (**Figure 1B-a**). The graph presented in (**Figure 1B-c**) shows the quantification of the relative 5-BP fluorescent intensities that are normalized to number of cells per image. Together, these data showed that hypoxia enhanced the expression and activity of TG2 and was time -dependent.

### Effect of Hypoxia on the Activity of SERCA2 and Expression of SERCA2 Serotonylation

**Figure 2:**
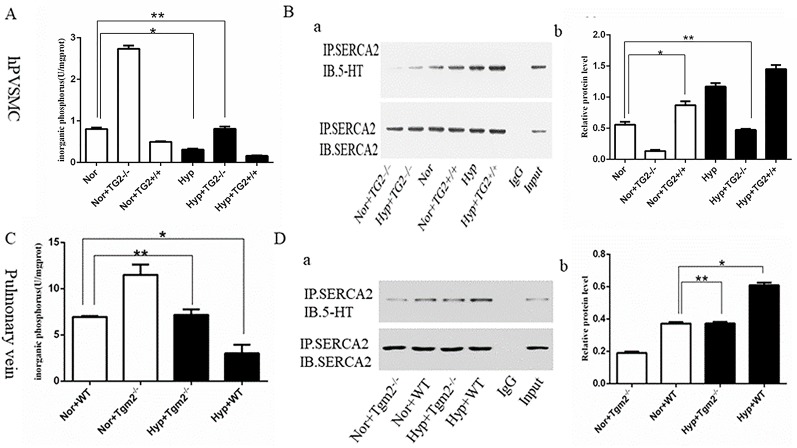
The inorganic phosphorus production and s-SERCA2 protein expression of human pulmonary vein smooth muscle cells (hPVSMCs) and the pulmonary vein. Cells were treated with Ca2+ (6.7 mM), 5-HT (1 mM), and hypoxia (1%) for 24 h. Mice were housed under hypoxic conditions (10% O2) for 6 weeks. All values are presented as the mean ± S.E.M. Inorganic phosphorus, IP; Hyp, hypoxia; Nor, normoxia; TG2−/−, TG2 gene silencing; TG2+/+, TG2 gene overexpression; WT, wild type mice; Tgm2−/−, vascular smooth muscle-specific TG2 knockout mice. **(A)** Analysis of inorganic phosphorus produced by six groups of hPVSMCs. Hypoxia significantly prevented cells from producing inorganic IP (n = 3, *p < 0.05 *vs.* normoxia). When compared to the normoxia group, silencing of the TG2 gene did not increase IP under hypoxia (n = 3, **p > 0.05 compared with normoxia). **(B)** Co-IP analyzing the expression of s-SERCA2 protein *in vitro*. Relative expression of s-SERCA2 protein. Hypoxia significantly increased s-SERCA2 protein expression on hPVSMCs (n = 3, *p < 0.05 *vs.* normoxia). When silencing TG2 gene, hypoxia did not affect s-SERCA2 protein expression under hypoxia (n = 3, **p > 0.05 compared with normoxia). **(C)** Analysis of inorganic phosphorus produced by WT and Tgm2−/− mice. WT mice produced more IP under normoxia than under hypoxia (n = 6, *p < 0.05 *vs*. normoxia). There no differences were observed in the production of IP between WT mice under normoxia and Tgm2−/− mice under hypoxia (n = 6, **p > 0.05 compared with WT mice under normoxia). **(D)** Co-IP analyzed expression of s-SERCA2 protein *in vivo*. Relative expression of s-SERCA2 protein. Hypoxia significantly increased s-SERCA2 protein expression (n = 6, *p < 0.05 *vs*. WT mice under normoxia). Hypoxia increased the expression of s-SERCA2 protein in WT mice, but did not affect the expression of s-SERCA2 protein in Tgm2^−/−^ mice (n = 6, **p > 0.05 compared with WT mice under normoxia).

*In vitro*, cells produced more organic phosphorus (IP) when the TG2 gene was silenced, whereas cells produced less organic phosphorus when the TG2 gene overexpressed under normoxia. In addition, our findings showed that hypoxia significantly prevented cells from producing inorganic phosphorus, which was regulated by TG2. When compared to the normoxia group, silencing of the TG2 gene did not increase the production of inorganic phosphorus under hypoxic conditions (**Figure 2A**). Next, we used co-IP to analyze the level of s-SERCA2 protein. Our data showed that the expression of s-SERCA2 protein was significantly increased when the TG2 gene was overexpressed, whereas the expression of s-SERCA2 protein decreased when the TG2 gene was silenced under normoxic conditions. When compared to the normoxia group, the expression of s-SERCA2 protein significantly increased under hypoxia, however the expression did not significantly increase when the TG2 gene was silenced under hypoxia. When comparing the two groups of cells regarding TG2 gene overexpression or TG2 gene silencing, we found that the activity of TG2 played a key role in serotonylation *in vitro* (**Figure 2B**). *In vivo*, Tgm2^−/−^ mice produced more organic phosphorus than WT mice under normoxic and hypoxic conditions, however there no differences were observed in the production of organic phosphorus between WT mice under normoxia and Tgm2^−/−^ mice under hypoxia (**Figure 2C**). The levels of SERCA2-s on the PV were detected by co-IP. The expression of s-SERCA2 protein in WT mice was higher when compared to that in TG2 mice under normoxia. Hypoxia increased the expression of s-SERCA2 protein in WT mice, but did not affect the expression of s-SERCA2 protein in Tgm2^−/−^ mice. Similarly, when comparing the two groups of WT mice or the two groups of Tgm2^−/−^ mice, we found that the activity of TG2 played a key role in serotonylation *in vivo* (**Figure 2D**). Combined, these findings showed that serotonylation of SERCA2 inhibited the activity of SERCA2, and hypoxia induced serotonylation of SERCA2, which was modulated by the key enzyme of TG2 *in vivo* and *in vitro*. Furthermore, the activity of TG2 may play a key role in SERCA2 serotonylation both *in vivo* and *in vitro*.

### Effect of SERCA2 Serotonylation on [Ca2+]i and Store-Operated Calcium Entry in Human Pulmonary Vein Smooth Muscle Cells

**Figure 3:**
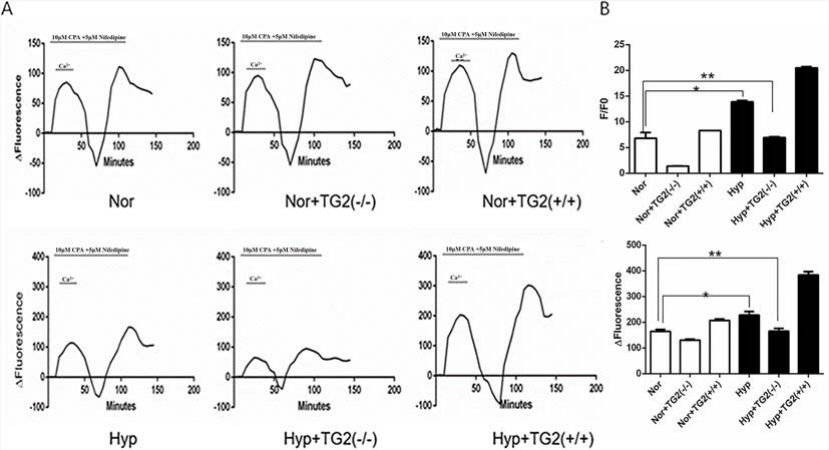
Effect of SERCA2 serotonylation on [Ca2+]i and store-operated calcium entry (SOCE) in human pulmonary vein smooth muscle cells (hPVSMCs). The ratio of the fluorescence intensity (F/F0) was used to compare [Ca2+]i. The difference in fluorescence intensity was used to compare SOCE. L-type voltage-dependent calcium channel (VDCC) antagonist, nifedipine. SERCA2 antagonist, cyclopiazonic acid (CPA). All values are presented as the mean ± S.E.M. **(A)** Time course curvilinear of time−Δfluorescence by GraphPad software to analyze SOCE. ΔFluorescence, the difference in fluorescence intensity between cells perfused with Hank’s Balanced Salt Solution (HBSS) and Ca2+-free HBSS. **(B)** Semi-quantitative analysis of intracellular basal [Ca2+]i by F/F0 (n = 3, *p < 0.05 compared with the normoxic group). **(C)** Histogram analysis of the peak-to-valley value of the time-fluorescence curve (n = 3, **p > 0.05 compared with normoxic group).

As shown in **Figure 3B**, hypoxia (1% O2) induced a marked increase in basal [Ca2+]i from 6.81 ± 1.14 to 13.88 ± 0.26. In the normoxia group, the cell basal [Ca2+]i decreased from 6.81 ± 1.14 to 1.40 ± 0.04 when the TG2 gene was silenced, and increased from 6.81 ± 1.14 to 8.30 ± 0.03 when the TG2 gene was overexpressed. In the hypoxia group, the cell basal [Ca2+]i decreased from 13.88 ± 0.26 to 6.95 ± 0.16 when the TG2 gene was silenced, and increased to 20.48 ± 0.25 when the TG2 gene was overexpressed. Thus, both groups showed a similar trend. Further comparison of the two groups showed that hypoxia did not significantly reduce the basal [Ca2+]i when the TG2 gene was silenced. Our data showed that nifedipine specifically blocked the L-type voltage-dependent calcium channel (VDCC). CPA promoted the release of calcium ions from the sarcoplasmic reticulum, causing a brief increase in [Ca2+]i that allowed for large amounts of calcium ions to flow into cells through the SOCE channel after restoring the extracellular calcium concentration. As shown in **Figures 3A, C**, semi-quantitative detection of SOCE by Δfluorescence [the difference in fluorescence intensity between cells that were perfused Hank’s Balanced Salt Solution (HBSS) and Ca2+-free HBSS] under normoxia and hypoxia. We established a time course curvilinear of time−Δfluorescence by GraphPad software to analyze dynamic changes in [Ca2+]i. Hypoxia induced a marked increase in the peak of time−Δfluorescence curve from 164.33 ± 7.64 to 228.00 ± 14.42. In the normoxia group, the peak Δ[Ca2+]i decreased from 164.33 ± 7.64 to 130.33 ± 4.51 when the TG2 gene was silenced, and increased to 207.00 ± 6.25 when the TG2 gene was overexpressed. In addition, in the hypoxia group, the peak Δ[Ca2+]i decreased from 228.00 ± 14.42 to 165.33 ± 11.01 when the TG2 gene was silenced, and increased to 383.67 ± 13.50 when the TG2 gene was overexpressed. Further comparison of the two groups showed that hypoxia did not significantly increase the calcium influx when the TG2 gene was silenced. Thus, these data showed that TG2-mediated SERCA2 serotonylation can promote calcium ion influx through SOCE.

### Effect of TRPC1 and TRPC6 on [Ca2+]i and Store-Operated Calcium Entry in Human Pulmonary Vein Smooth Muscle Cells Under Hypoxia

**Figure 4:**
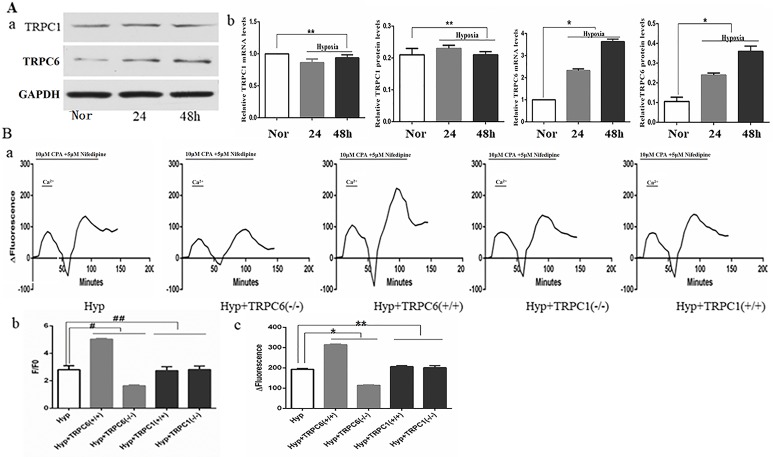
Expression of TRPC1, TRPC6, and the effect of TRPC1 and TRPC6 on [Ca2+]i and store-operated calcium entry (SOCE) in human pulmonary vein smooth muscle cells (hPVSMCs) under hypoxia. L-type voltage-dependent calcium channel (VDCC) antagonist, nifedipine. SERCA2 antagonist, cyclopiazonic acid (CPA). Nor, normoxia; Hyp, hypoxia; TRPC(−/−), TRPC gene silencing; TRPC(+/+), TRPC overexpression. ΔFluorescence, difference in fluorescence intensity between cells perfused with Hank’s Balanced Salt Solution (HBSS) and Ca2+-free HBSS. All values are presented as the mean ± S.E.M. **(A**-a**)** TRPC1 and TRPC6 protein bands and the level of TG2 protein. **(A**-b**)** The relative level of TRPC1 messenger RNA (mRNA) and protein (n = 3, **p > 0. 05 as compared to the normoxic group). **(A**-c**)** The relative level of TRPC6 mRNA and protein (n = 3, *P < 0. 05 as compared to the normoxic group). **(B)** The ratio of fluorescence intensity (F/F0) was used to compare [Ca2+]i. Time-Δfluorescence curve was used to compare SOCE. **(B**-a**)** Time course curvilinear of time-Δfluorescence by GraphPad software to analyze SOCE. **(B**-b**)** Semi-quantitative analysis of [Ca2+]i by F/F0 (n = 3, ^#^p < 0.05 as compared to hypoxia group, ^##^p > 0.05 as compared to the hypoxia group). **(B**-c**)** Histogram analysis of the peak-to-valley value of the time-fluorescence curve (n = 3, *p < 0.05 as compared to hypoxia group).

Many studies have shown that hypoxia increased expression of TRPC6, not TRPC1, on pulmonary artery smooth muscle cells (Lu et al., 2008; Xia et al., 2014; Wang et al., 2016). When compared to the control group, the expression of TRPC6 mRNA and protein was significantly increased and time-dependent under hypoxia, whereas the expression of TRPC1 mRNA and protein was not significantly increased or decreased under hypoxia (**Figure 4A**). As one of the main channels mediating the extracellular calcium influx on the cell membrane, the TRPC channel was an indispensable molecule during the formation of chronic hypoxic pulmonary hypertension (CHPH). We analyzed [Ca2+]i and SOCE by silencing and overexpression of the TRPC1 and TRPC6 genes under hypoxia. Semi-quantitative analysis of basal [Ca2+]i by F/F0 and SOCE was performed by time-Δfluorescence curve under hypoxia. As shown in **Figure 4B-b**, the basal [Ca2+]i increased from 2.81 ± 0.29 to 5.04 ± 0.05 after TRPC6 gene overexpression, but decreased to 1.64 ± 0.06 after TRPC6 gene silencing. The basal [Ca2+]i of silencing and overexpressing of the TRPC1 gene were 2.82 ± 0.25 and 2.73 ± 0.29, respectively. When compared with normal cells, [Ca2+]i was not significantly different when the TRPC1 gene was silenced or overexpressed. Next, we measured the peak of time-Δfluorescence curve to analyze SOCE. As shown in **Figure 4B-a, c**, the peak fluorescence increased from 193.00 ± 4.00 to 314.67 ± 2.89 after TRPC6 gene overexpression, and decreased to 114.33 ± 2.31 after TRPC6 gene silencing. The peak fluorescence of silencing and overexpressing TRPC1 gene were 200.67 ± 10.60 and 206.00 ± 5.00, respectively. In fact, silencing or overexpressing TRPC1 did not effect peak fluorescence. Combined, these data showed that hypoxia activated TRPC6-mediated SOCE to promote the extracellular calcium influx.

### Effect of Cyclopiazonic Acid and PST-2744 on Human Pulmonary Vein Smooth Muscle Cell Proliferation, Apoptosis, Migration, [Ca2+]i, Store-Operated Calcium Entry, and Cell Phenotype

#### Proliferation, Apoptosis, and Migration

**Figure 5:**
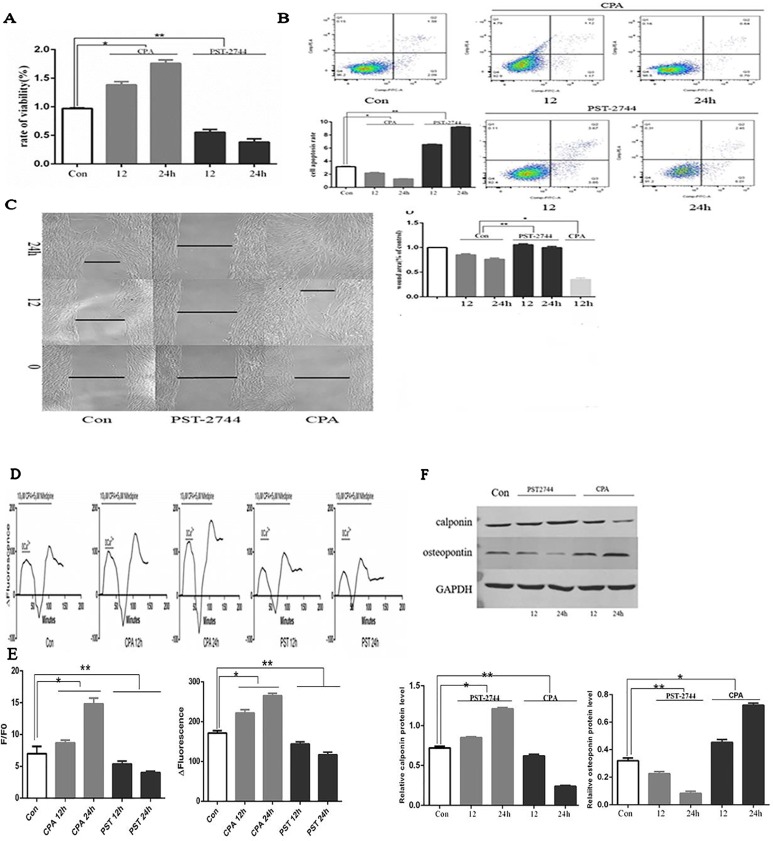
Effect of cyclopiazonic acid (CPA) and PST-2744 on cell proliferation, apoptosis, migration, [Ca2+]i, store-operated calcium entry (SOCE), and cell phenotype. PST, PST-2744; Con, control. 12, 12 h. 24, 24 h. ΔFluorescence, difference in fluorescence intensity between cells perfused with Hank’s Balanced Salt Solution (HBSS) and Ca2+-free HBSS. All values were presented as the mean ± S.E.M. **(A)** Rate of proliferation measured by Cell Counting Kit (CCK)-8 assay. CPA promoted cell proliferation in a time-dependent manner. PST-2744 suppressed cell proliferation in a time-dependent manner (n = 3, *p < 0.05 as compared to the control group, **p < 0.05 as compared to the control group). **(B)** Apoptosis rate measured by flow cytometry. CPA suppressed cell apoptosis in a time-dependent manner. PST-2744 promoted cell apoptosis in a timely fashion. Quantitative analysis of the apoptosis rate (n = 3, *p < 0.05 as compared to the control group, **p < 0.05 as compared to the control group). **(C)** Wound healing assay for human pulmonary vein smooth muscle cells (hPVSMCs). CPA promoted wound healing, whereas PST-2744 prevented wound healing. **(C)** Quantification of wound-healed area of hPVSMCs (n = 3, *p < 0.05 as compared to the control group, **p < 0.05 as compared to the control group). **(D)** Time-Δfluorescence curve was used to compare SOCE. Time course curvilinear of time-Δfluorescence by GraphPad software to analyze SOCE. Histogram analysis of the peak-to-valley value of the time-fluorescence curve (*p < 0.05 as compared to the control group, **p < 0.05 as compared to the control group). **(E)** The ratio of fluorescence intensity (F/F0) was used to compare [Ca2+]i. Semi-quantitative analysis of [Ca2+]i by F/F0 (n = 3, *p < 0.05 compared with the control group, *p < 0.05 compared with the control group). **(F)** Western blot analysis for the expression of calopnin and osteopontin protein. PST-2744 suppressed the expression of osteopontin protein, but promoted expression of calopnin protein. CPA suppressed the expression of calopnin protein, but promoted expression of osteopontin protein. Quantitative analysis of the relative expression of calopnin and osteopontin protein (n = 3, *p < 0.05 compared with the control group, **p < 0.05 compared with the control, *p < 0.05 compared with the control, **p < 0.05 compared with the control group).

When compared to the control group, CPA promoted proliferation of hPVSMCs in a time-dependent manner, but PST-2744 inhibited proliferation of hPVSMCs in a timely fashion (**Figure 5A**). Secondly, examined the rate of cell apoptosis by using flow cytometry. When compared to the control group, CPA inhibited apoptosis of hPVSMCs in a time-dependent manner, however PST-2744 promoted apoptosis of hPVSMCs in a time-dependent manner (**Figure 5B**). Thirdly, we analyzed migration of cells by using Culture-Insert 2 Well (ibidi, German). Compared to control group, CPA promoted cell migration and resulted in a reduced wound left by the insert. However, PST-2744 prevented cells migration and resulted in a residual wound by the insert (**Figure 5C**). These data showed that CPA inhibited cell apoptosis, and promoted cell proliferation and migration, whereas PST-2744 promoted cell apoptosis, and inhibited cell proliferation and migration.

#### [Ca2+]i and Store-Operated Calcium Entry

SERCA is an ion pump that takes up calcium ions from the cytoplasm of the endoplasmic reticulum. The decreased expression or activity of SERCA2 was the main cause of persistent SOCE. Time-Δfluorescence curve (**Figure 5E**) showed that the normal [Ca2+]i was 6.97 ± 1.16. Moreover, the [Ca2+]i of cells treated with CPA for 12 and 24 h was 8.69 ± 0.43 and 14.82 ± 0.88, respectively. In addition, the [Ca2+]i of cells treated with PST-2744 for 12 and 24 h was 5.36 ± 0.46 and 4.03 ± 0.19, respectively. As shown in **Figure 5D**, the peak of time-Δfluorescence curve was measured to analyze SOCE. The normal peak fluorescence was 171.33 ± 6.11. The peak fluorescence of cells treated with CPA for 12 and 24 h were 221.67 ± 8.62 and 265.00 ± 6.00, respectively, and the peak fluorescence the of cells treated with PST-2744 for 12 and 24 h was 143.67 ± 5.69 and 116.67 ± 6.51, respectively. Combined, these data showed that inhibition of the SERCA2 activity promoted extracellular calcium influx in a time-dependent manner, whereas and increase in SERCA2 activity decreased the extracellular calcium influx in a time-dependent manner.

#### Cell Phenotype

Unlike skeletal muscle and cardiomyocytes, vascular smooth muscle cells (VSMCs) are non-terminally differentiated cells with a strong plastic phenotype. The VSMCs of normal adult animals were mainly contractile phenotypes. Under the influence of various stimulating factors, the phenotype of VSMCs can be transformed from a differentiated phenotype with a contractile function to a dedifferentiated phenotype with a strong proliferation and migration ability. In this study, we focused on contractile phenotype protein markers, calopnin, and secreted protein marker, osteopontin, which were evaluated by Western blot analysis. PST-2744 suppressed the expression of osteopontin protein, but promoted the expression of calopnin protein. In addition, CPA suppressed the expression of calopnin protein, but promoted expression of osteopontin protein (**Figure 5F**). These data showed that CPA promoted transformation from a differentiated phenotype to a dedifferentiated phenotype, and PST-2744 promoted transformation from a dedifferentiated phenotype to a differentiated phenotype.

### TG2 Reverses Hypertension of the Right Ventricle and Remodeling Vascular

**Figure 6:**
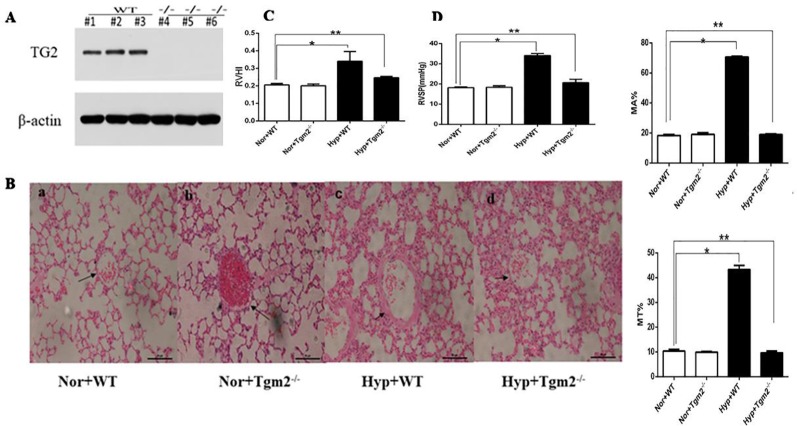
TG2 reverses right ventricle pressure and vessel remodeling. WT, wild type; Hyp, hypoxia; Nor, normoxia; Tgm2−/−, TG2 gene knockout. MT% = 100×(medial layer thickness)/(vessel semidiameter) and area [MA% = 100×(cross-sectional medial layer area)/(total cross-sectional vessel area)]. All values are denoted as the mean ± S.E.M. **(A)** Homozygous mice-SM22α-Cre+. Western blot analysis of pulmonary veins using an anti-TG2 polyclonal antibody. **(B)** Hematoxylin-eosin (H&E) staining in mice pulmonary vessels. No difference in vessel wall thickness was observed between WT and Tgm2^−/−^ mice under normoxia. The wall thickness of pulmonary vessels significantly increased in WT mice after 6 weeks of hypoxia (n = 6, *P > ;0.05 compared with under normoxia, **P > 0.05 compared with under normoxia). **(C**, **D)** The measurement of right ventricular systolic pressure (RVSP) and right ventricular hypertrophy index (RVHI) in wild-type (WT) and TG2 knockout mice were treated with normoxia and hypoxia (5% O2, 6 weeks) (n = 6, *P > ;0.05 compared with under normoxia, **P > 0.05 compared with under normoxia).

*In vivo*, as shown in **Figure 6A**, based on mouse tail genetic identification, heterozygous and SM22α-Cre− was considered wild-type (WT), homozygous and SM22α-Cre+ was considered a Tgm2 complete knock-out (Tgm2−/− type). Three WT and Tgm2−/− mice were randomly selected to identify the expression of TG2 protein by Western blot (WB) analysis, which indicated that TG2 protein was normally expressed in the PV of WT mice but not in that of Tgm2−/− mice. As shown in **Figures 6C**, **D** we measured the right ventricular systolic pressure (RVSP) of mice by using a closed-chest insertion into the right ventricle (RV) and a xiphocostal angle approach under hypoxia for 6 weeks. We found that the RVSP of Tgm2−/− mice and WT mice was 18.15 ± 0.45 mmHg and 18.35 + 0.76 mmHg, respectively, under normoxia, which was not significantly different between groups. The RVSP of WT mice increased from 18.15 ± 0.45 mmHg to 34.05 ± 0.99 mmHg after hypoxia exposure for 6 weeks, and we noticed some differences between WT mice under normoxia and Tgm2−/− mice under hypoxia. The RVSP of Tgm2−/− mice under hypoxia 22.79 ± 6.79 mmHg was slightly higher when compared to that of WT mice under normoxia, however no statistical significance was achieved. After measurement of RVSP, heart and lung tissues were collected. Weights of the RV and the left ventricle (LV) plus interventricular septum (LV+S) were measured separately. The RVHI of Tgm2−/− mice and WT mice were 0.20 ± 0.010 and 0.21 + 0.009 under normoxia, respectively. Moreover, the RVHI of Tgm2−/− mice and WT mice were 0.25 ± 0.063 and 0.34 + 0.055 under hypoxia. The trend of RHVI was consistent with that of RVSP. As shown in **Figure 6B**, hematoxylin and eosin (H&E) staining showed that MT% of Tgm2−/− mice and WT mice were 9.90 ± 0.30 and 10.37 ± 0.67, and MA% of Tgm2−/− mice and WT mice were 19.13 ± 1.00 and 18.40 ± 0.62 under normoxia respectively. The MT% of Tgm2−/− mice and WT mice were 9.63 ± 0.70 and 43.30 ± 1.67, and MA% of Tgm2−/− mice and WT mice were 8.97 ± 0.47 and 70.67 ± 0.50 under hypoxia respectively. The data showed that there was no significant difference in vessel wall thickness between Tgm2−/− and WT mice under normoxia. However, vascular wall thickness of WT mice significantly increased after hypoxic treatment, whereas vascular wall thickness of Tgm2−/− mice was not significantly increased after hypoxic treatment. Together, these data showed that TG2 reversed hypertension of the right ventricle and vascular remodeling.

## Discussion

Here, we revealed a novel mechanism in which TG2 alleviated hypoxia-induced pulmonary vein remodeling. Our findings demonstrated that the activity of SERCA2 was regulated by a new post-translational modification (PTM) “serotonylation” and SERCA2 serotonylation inhibited activity of SERCA2. Cytoplasmic Ca2+ storage was depleted when the activity of SERCA2 was inhibited, then TRPC6 was activated which further increased SOCE to lead to a continuous increase of intracellular calcium, consequently promoting proliferation and migration of hPVSMCs. Moreover, we demonstrated that the activity of SERCA2 played an important role on the biological behavior of hPVSMCs.

It is well known that 5-HT plays an important role on the development of HPAH. In order to understand the molecular mechanism involved, most studies focused on the 5-HT transporter and 5-HT receptor. For example, some evidence indicated that 5-HT-related stimulation of pulmonary artery smooth muscle cell (PASMC) proliferation required 5-HT internalization through the 5-HT transporter (5-HTT) (Lee et al., 1991; Lee et al., 1998; Lee et al., 1999; Eddahibi et al., 2001; Abid et al., 2012). In addition, 5-HT2A and 5-HT2B were also involved in hypoxia-induced pulmonary artery remodeling and proliferation of human pulmonary artery smooth muscle cells (hPASMC) (Keegan et al., 2001; Welsh et al., 2004; Maclean and Dempsie, 2010; Occhiogrosso et al., 2012). New insight regarding the possible mechanism by which intracellular 5-HT might exert an intracellular effect stems from our *in vitro* studies with SMCs, which showed that 5-HT serotonylates (transaminates) take up intracellular proteins through the enzyme transglutaminase (TGase) (Liu et al., 2011).

As previously mentioned, transglutaminase2 (TG2) is a ubiquitous multifunctional protein that catalyzed the post-translational modification of proteins *via* a calcium-dependent transglutamidation reaction. Modification of TG2 substrate proteins by transamidation has been shown to be important in cell survival, apoptosis, and cytoskeleton organization (Watts et al., 2009; Kumar and Mehta, 2012; Gundemir et al., 2013; Penumatsa and Fanburg, 2014). Moreover, its expression is finely regulated at the transcriptional level by cytokines, retinoids, NFκB, and by inflammatory and hypoxic stimuli (Ientile et al., 2007; Nurminskaya and Belkin, 2012; Eckert et al., 2014). In fact, as early as in 2003, Walther et al. firstly demonstrated that serotonin (5-HT) was transamidated to small GTPases by transglutaminases during activation and aggregation of platelets, rendering these GTPases constitutively active, and provided evidence for a receptor-independent signaling mechanism, termed here as “serotonylation” for the first time (Walther et al., 2003). Several studies have demonstrated that the serotonylation of RhoA, fibronectin, and smooth muscle b-actin all play important roles in aortic vascular contractility (Guilluy et al., 2007; Guilluy et al., 2009; Liu et al., 2011).

It is well known that the ryanodine receptor (RyR) and SERCA play an important role in hypoxia-induced pulmonary vascular remodeling by regulating calcium influx. Chronically high levels of intracellular calcium in pulmonary SMCs trigger signaling pathways that allow cellular proliferation, migration, and dedifferentiation, all of which are factors that contribute to hypertrophic vascular remodeling (Kuhr et al., 2012), and SERCA2 was a key modulator of calcium cycling in both cardiomyocytes and vascular SMCs (Kawase and Hajjar, 2008). Hypoxia reduced SERCA2 activity, however its mechanism of action was unclear, involving a variety of accessory proteins and kinases, and SERCA2 had multiple PTM of protein sites. PTM was an important way to regulate its activity (Vangheluwe et al., 2005). More importantly, once the activity was inhibited, it was difficult to reverse (Tong et al., 2010). Our study firstly proposed a new PTM of SERCA2, SERCA2 serotonylation. We demonstrated that hypoxia enhanced SERCA2 serotonation, expression, and activity of TG2 both *in vivo* and *in vitro*. Conversely, hypoxia inhibited the activity of SERCA2 *in vivo* and *in vitro*. To further investigate the effect of serotonation on the activity of SERCA2, we established vascular smooth muscle-specific TG2 knockout (Tgm2−/−) mice, overexpressed, and silenced TG2 gene on cells, and the results demonstrated that TG2-mediated serotonation inhibited SERCA2 activity under hypoxia. As mentioned earlier, our results confirmed that TG2 was a ubiquitous multifunctional protein that catalyzed the PTM, which was consistent with the increased activity of TG2 that promoted fibronectin (sFn) serotonylation in the sera of humans with pulmonary arterial hypertension (PAH) and in the sera and lungs of experimental rodent models of PH (Penumatsa et al., 2014a). In fact, there was a potential cross-talking between serotonylation and other relevant signal pathway. According to Wu et al., during early reperfusion, the ROS/JAK2/STAT3 pathways play a crucial role in i) IHH (intermittent hypobaric hypoxia)-maintained intracellular CA(2+) homeostasis by improving postischemic SERCA2 activity, by increasing SR Bcl-2 and its interaction with SERCA2; and ii) the IHH-improved mitochondrial functioning (Yuan et al., 2009). Oxidative stress influences various proteins. Oxidative stress influences various proteins and biological processes, and there was also cross-interaction between oxidative stress and SUMOylation (Rigato et al.,2002; Feligioni et al., 2011; Leitao et al., 2011; Wu et al., 2015). The intensity of SUMOylation of many proteins was affected by oxidative stress (Shishido et al., 2008; Pandey et al., 2011; Shrivastava et al., 2014; Sahin et al., 2014). SUMOylation enhances the stability and activity of SERCA2a (Feligioni and Nistico, 2013). Upgrading the intensity of SERCA2a-SUMOylation increases the protein level of SERCA2a and improves cardiac function in animal models with heart failure (HF) (Feligioni and Nistico, 2013). Although the relation between oxidative stress and SERCA2a-SUMOylation still needs to be investigated, Jing Yao et al. suggested the possible role of SERCA2a-SUMOylation on the obesity-induced cardiac dysfunction and PA-induced cardiomyocyte dysfunction (Kho et al., 2011).

It was generally believed that SOCE was a small and persistent calcium influx, often associated with chronic hypoxic pulmonary vascular remodeling (Yuan et al., 2009; Wu et al., 2015). We found that TRPC6, not TRPC1 was highly expressed in hPVSMCs after hypoxia treatment, and basal [Ca2+]i and SOCE were increased and decreased when the TRPC6 gene silenced and overexpressed, respectively. However, silencing or overexpression of the TRPC1 gene did not have an effect on [Ca2+]i and SOCE. These results suggested that CH may increase SOCE and [Ca2+]i through activating the TRPC6 channel on hPVSMCs, however the mechanism was not clear. Our study highly suggested that SERCA2 serotonination may be a new mechanism for hypoxia-induced imbalance of intracellular calcium ions. We further demonstrated that the activity of SERCA2 was closely related to proliferation, migration, and phenotype of cells by using an inhibitor and agonist of SERCA2. Furthermore, we demonstrated that the activity of TG2 played a key role in serotonylation *in vivo* and *in vitro*.

Finally, we analyzed the vascular remodeling of Tgm2−/− mice under hypoxia. The index of morphology and hemodynamics of WT mice was consistent with pathological features of CHPH under hypoxia for 6 weeks. However, after the same treatment of hypoxia, the above-mentioned indexes of Tgm2−/− mice did not significantly increase, although it did not reach the level of WT mice under normoxia. Therefore, we speculated that TG2 can reverse pulmonary vascular remodeling, which provided a novel target and orientation for treatment of CHPH. A limitation of the current experimental design was the low replicates (n = 3) in the cell-based experiments, albeit statistical significance was reached.

## Conclusions

**Figure 7:**
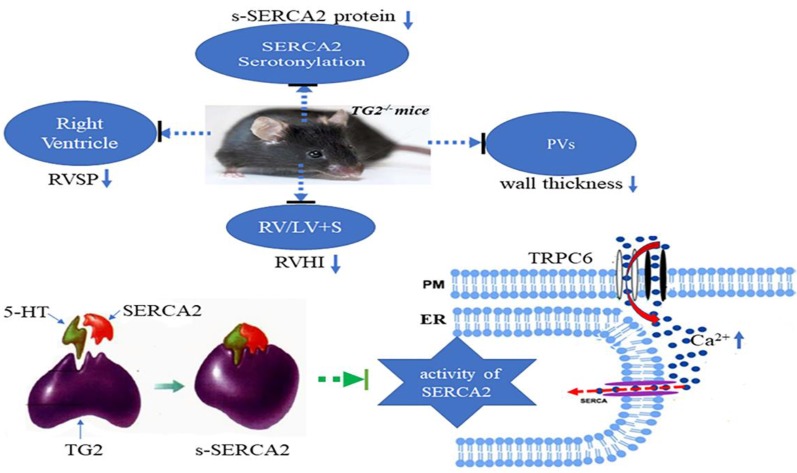
A model for TG2-mediated SERCA2 serotonylation on hypoxic pulmonary vein remodeling. Upon depletion of ER Ca2+ stores, store-operated calcium entry (SOCE) channels and TRPC6 channels promoting entry of Ca2+ across the plasma membrane (PM). Hypoxia promoted TG2-mediated SERCA2 serotonylation, leading to inhibition of SERCA2 activity, which further increased the calcium influx through the TRPC6 channel, which eventually resulted in excessive cell proliferation, migration, and blockage of apoptosis, thereby promoting pulmonary vascular remodeling. Right ventricular systolic pressure (RVSP), right ventricular hypertrophy index (RVHI), and wall thickness of pulmonary veins (PVs) of Tgm2−/− mice did not significantly increase under hypoxia for 6 weeks.

We firstly demonstrated that hypoxia promoted TG2-mediated SERCA2 serotonylation, leading to inhibition of SERCA2 activity, which further increased the calcium influx through the TRPC6 channel, which eventually resulted in excessive cell proliferation, migration, and blockage of apoptosis, thereby promoting pulmonary vascular remodeling (**Figure 7**).

## Data Availability Statement

The raw data supporting the conclusions of this manuscript will be made available by the authors, without undue reservation, to any qualified researcher.

## Ethics Statement

All of the procedures carried out on animals were approved by the Animal Care and Use Committee of Nanjing Medical University, under animal protocol number IACUC11445. All protocols concerning the use of patient samples in this study were approved by the Ethics Committee of Zhongda Hospital, Southeast University.

## Author Contributions

CT and DW designed the study, and BL wrote the manuscript with support from EL, JH, YQ, GY, and QW. BL performed the *in vitro* experiments and the *in vivo* experiments. BL contributed to data interpretation and manuscript preparation. All authors read and approved the final manuscript.

## Funding 

This research was supported by the National Natural Science Foundation of China (Grant Number 81670237) and the Changzhou Health Commission of Science and Research (Grant Number QN201713).

## Conflict of Interest

The authors declare that the research was conducted in the absence of any commercial or financial relationships that could be construed as a potential conflict of interest.

## Abbreviations

CAP, cyclopiazonic acid; SOCE, store-operated calcium entry; [Ca2+]i, intracellular Ca2+ concentration; SERCA, sarco-endoplasmic reticulum Ca2+ ATPase; s-SERCA2, SERCA2 serotonination; TG2, transglutaminases2; PAH, pulmonary arterial hypertension; RVSP, right ventricular systolic pressure; RVHI, right ventricular hypertrophy index; hPVSMC, human pulmonary venous smooth muscle cell; TRPC, transient receptor potential cation channel; 5-HT, serotonin, 5-hydroxytryptamine; WT, wild type; Tgm2−/−, TG2 gene knockout.
