# Supplementary material for: Role of TG2-Mediated SERCA2 Serotonylation on Hypoxic Pulmonary Vein Remodeling
Source: Front Pharmacol. 2020 Feb 11;10:1611. doi: 10.3389/fphar.2019.01611 (PMC7026497; doi:10.3389/fphar.2019.01611)
Supplement: Supplementary file 1 [file DataSheet_1.docx]

**How much serotonylation contributes to SERCA2 activity/function**

5-HT is involved in the process of SERCA2 serotonylation. We investigated the effect of serotonination on the activity and function of SERCA2 by regulating 5-HT concentration and the incubation time. Inorganic phosphorus production was measured to determine the SERCA2 activity by using a commercially available kit. In addition, SERCA2 function was measured based on [Ca2+]i by using Fluo-4 AM. Firstly, hPVSMCs were incubated with 1 mM 5-HT for different timepoints (6, 12, and 24 hours) under both normoxia and hypoxia. Compared with 24 hours group, both normoxia and hypoxia promotes inorganic phosphorus production. While there was no difference in inorganic phosphorus production between 6 hours and 12 hours under hypoxia, deceased inorganic phosphorus production was observed at 24 hours (Fig. A). As shown in Figure B, basal [Ca2+]i is decreased from 6.81± 0.43(24h) to 4.37± 0.76(12h), and further reduced to 2.77±0.62(6h) under normoxia, the similar trend was observed under hypoxia. As shown in Figure C, peak of time－Δfluorescence curve deceased from 181.0±9.54(24h) to 153.00±6.56(12h), and further reduced to 109.33±7.37(6h) under normoxia, the same trend was also seen under hypoxia. Based on the above results, we concluded that hypoxia did not affect basal [Ca2+]i and SOCE at 6 hours and 12 hours. Thus, cells were treated with 1 mM exogenous 5-HT only for 24 hours can activate the physiological process of SERCA2 serotonylation. Secondly, hPVSMCs were incubated with different 5-HT concentrations (0.01-1 mM) for 24 hours. As mentioned earlier, we have determined that cells were treated with a certain concentration of 5-HT for enough time to activate SERCA2 serotonylation. As shown in Figure D, cells exposed to 0.1mM and 0.01mM 5-HT did not affect inorganic phosphorus production under hypoxia, despite cells were treated with 5-HT for 24h; however, hypoxia significantly reduced inorganic phosphorus production at 1mM 5-HT. As shown in Figure E, basal [Ca2+]i was significantly decreased from 6.94± 0.42(1mM) to 5.05± 0.84(0.1mM), and further reduced to 2.73±0.54(0.01mM) under normoxia, and 13.22±1.09(1mM) to 10.70±0.70(0.1mM) and then reduced to 7.36±0.42(0.01mM) under hypoxia. As shown in Figure F, peak of time－Δfluorescence curve deceased from 162.67±7.77(1mM) to 134.67±4.93(0.1mM) then reduced to 106.67±8.96(0.01mM) under normoxia, and 241.00±11.53(1mM) to 154.33±10.02(0.1mM) and then reduced to 135.33±8.08(0.01mM) under hypoxia. Based on the above results, we concluded that hypoxia did not affect basal [Ca2+]i and SOCE at 0.1mM 5-HT and 0.01mM 5-HT. Together, these data suggested that treatment of cells with 1 mM exogenous 5-HT for 24 hours may activate the physiological process of SERCA2 serotonylation under normoxia, and hypoxia may decrease the activity of SERCA2.


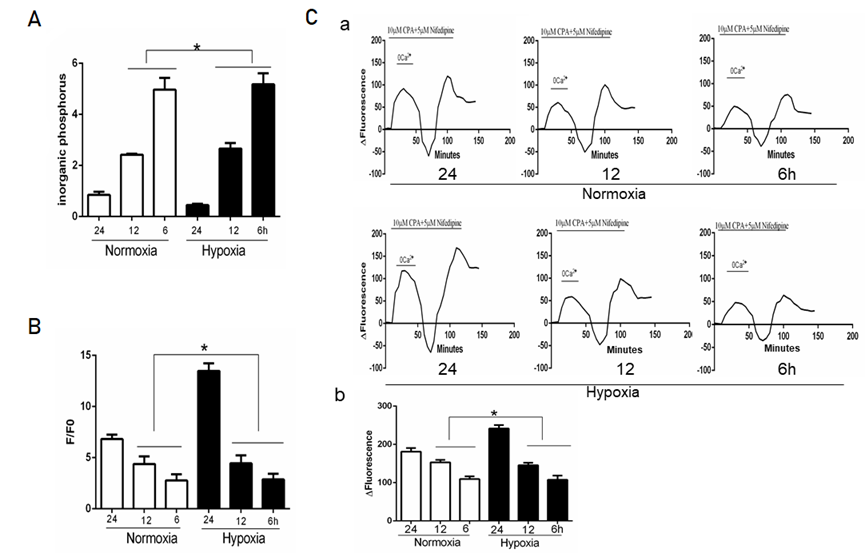


Figure. Effect of SERCA2 serotonylation on [Ca2+]i and SOCE in HPVSMCs. The ratio of the fluorescence intensity (F/F0) was used to compare [Ca2+]i. The difference in fluorescence intensity was used to compare SOCE. Cells were treated with Ca2+(6.7mM), 5-HT(1mM,0.1mM,0.01mM) under normoxia and hypoxia (1%) for 24h, and Ca2+(6.7mM) ,1mM 5-HT for 6,12 ,24 hours. All values are presented as the mean ± S.E.M. (A, C) Analysis of inorganic phosphorus produced by six groups of cells (*p>0.05 compared with cell under normoxia). (B, D) Semi-quantitative analysis of intracellular basal [Ca2+]i by F/F0. (*p>0.05 compared with the normoxia group). (E,F)Timecourse curvilinear of time－Δfluorescence by Graphpad software to analyze SOCE. Δfluorescence, the difference in fluorescence intensity between cells perfused with HBSS and Ca2+-free HBSS. Histogram analysis of the peak-to-valley value of the time-fluorescence curve (*p>0.05 compared with cell under normoxia).
