# Supplementary material for: Role of TG2-Mediated SERCA2 Serotonylation on Hypoxic Pulmonary Vein Remodeling
Source: Front Pharmacol. 2020 Feb 11;10:1611. doi: 10.3389/fphar.2019.01611 (PMC7026497; doi:10.3389/fphar.2019.01611)
Supplement: Supplementary file 2 [file DataSheet_2.docx]

1. Original pictures of Osteopomtin, calponin and GDPAH protein expression


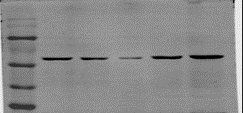
 osteopomtin


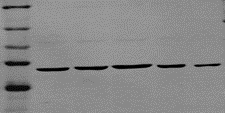
 calponin


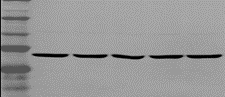
 GDPAH

1. Original pictures of knockdown and overexpression TG2, TRPC1 and TRPC6 gene by using RT-PCR and WB

Con TG2-/- TG2-/- TG2-/- TG2+/+ TG2+/+ TG2+/+


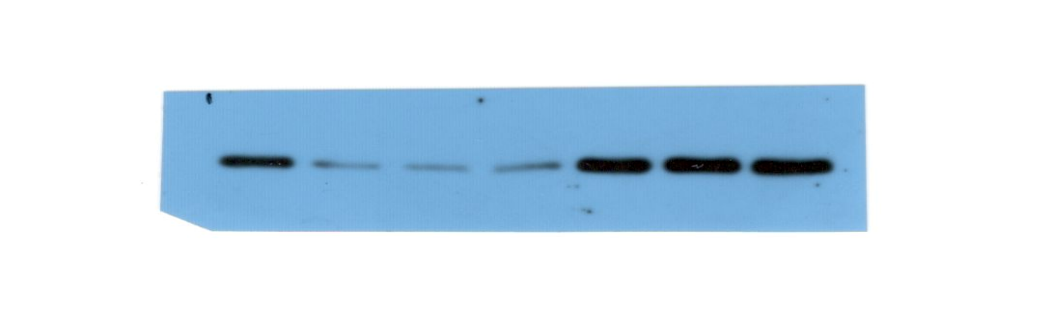
TG2


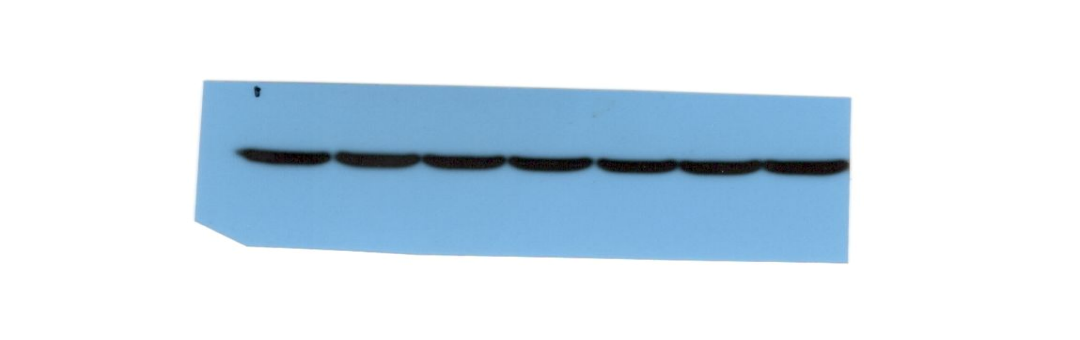
β-actin

TG2 protein level





TG2 mRNA level

Con TRPC1-/- TRPC1-/- TRPC1-/- TRPC1+/+ TRPC1+/+ TRPC1+/+


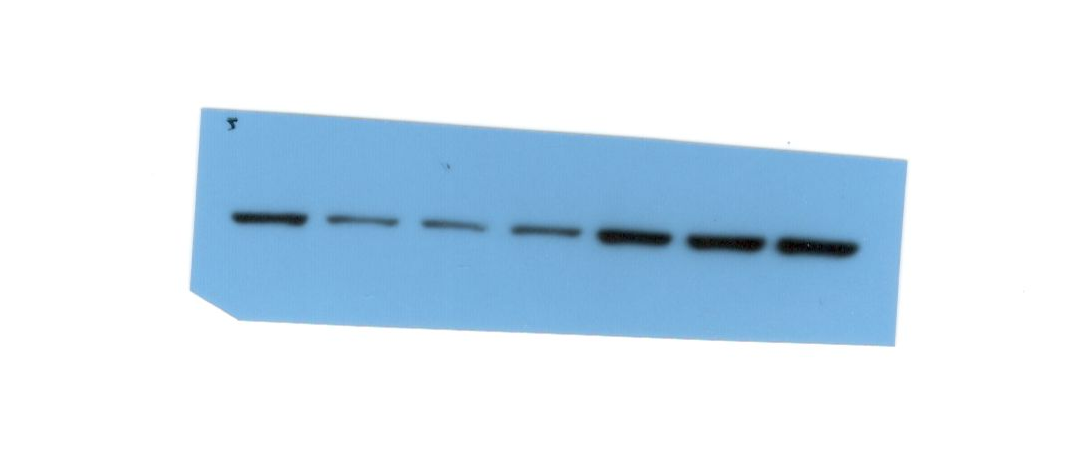
 TRPC1


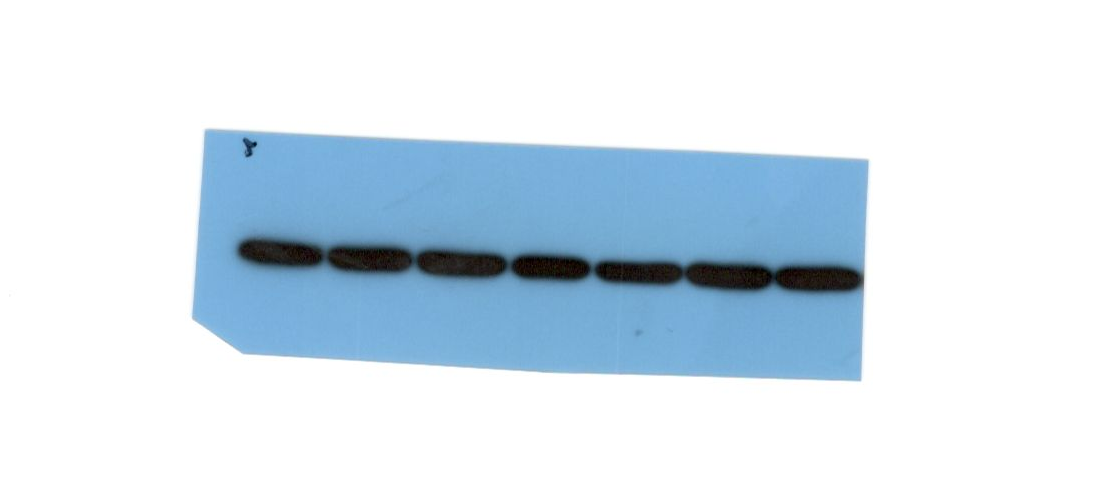
β-actin

TRPC1 protein level





TRPC1 mRNA level

Con TRPC6-/-TRPC6-/-TRPC6-/- TRPC6+/+ TRPC6+/+ TRPC6+/+


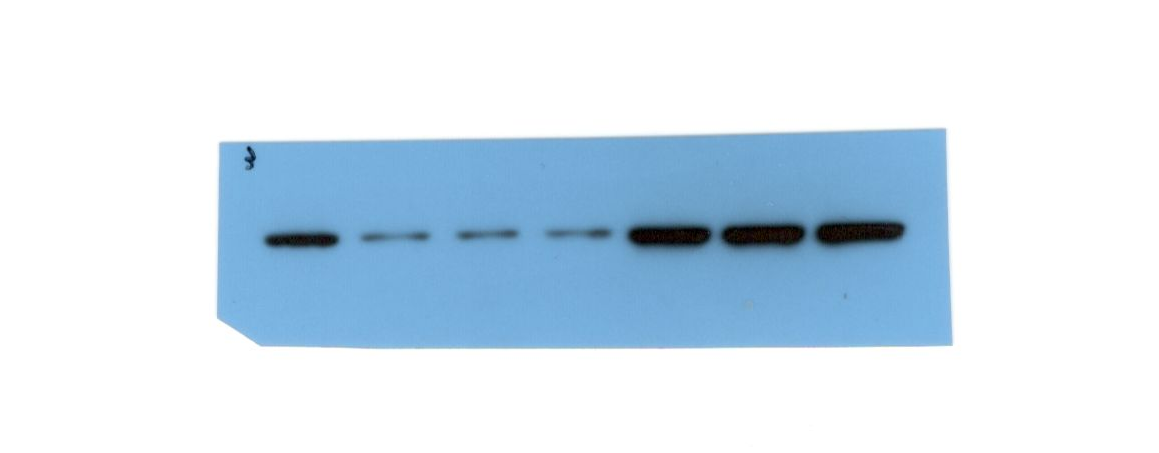
TRPC6


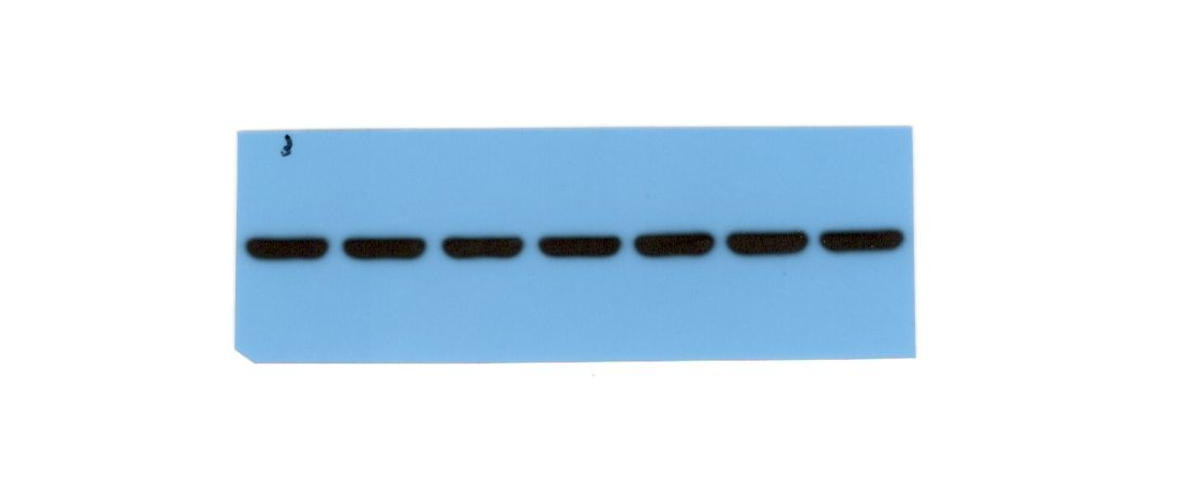
β-actin

TRPC6 protein level





TRPC6 mRNA level
