# Supplementary material for: Role of TG2-Mediated SERCA2 Serotonylation on Hypoxic Pulmonary Vein Remodeling
Source: Front Pharmacol. 2020 Feb 11;10:1611. doi: 10.3389/fphar.2019.01611 (PMC7026497; doi:10.3389/fphar.2019.01611)
Supplement: Supplementary file 3 [file DataSheet_3.docx]

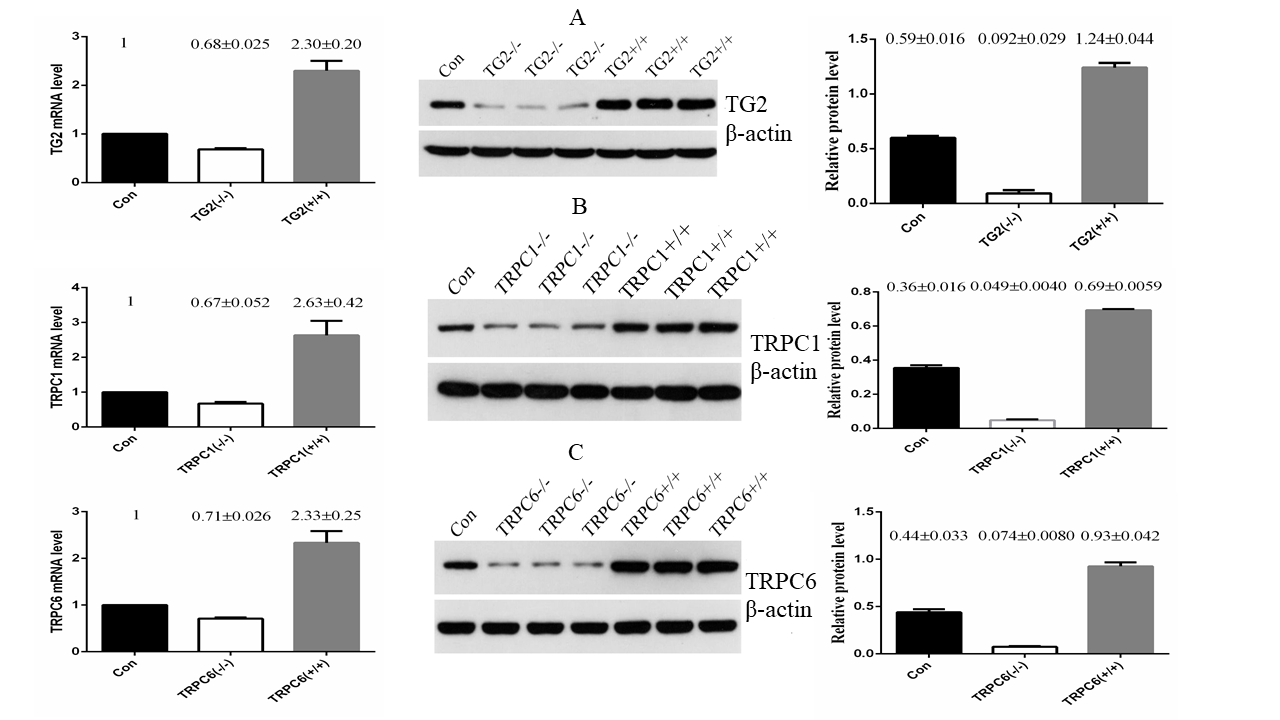


Silencing or overexpression TG2/TRPC1/TRPC6 gene efficiency analysis on hPVSMCs. Gene silencing used Lipofectamine^TM^ RNAiMAX Transfection Reagent, whose main component was liposome. Gene overexpression used recombinant adenovirus as a vector. Cells were incubated with siRNA-Lipofectamine^TM^ RNAiMAX Transfection Reagent complex for 8 hours and recombinant adenovirus for 12 hours, respectively. The expression of TG2/TRPC1/TRPC6 were detected by RT-PCR and WB when cells were treated with siRNA and recombinant adenovirus for 48 and 72 hours, respectively. Con. Control; TG2(-/-)/TRPC1(-/-)/TRPC6(-/-):TG2/TRPC1/TRPC6 gene silencing; TG2(+/+)/TRPC1(+/+)/TRPC6(+/+):TG2/TRPC1/TRPC6 gene overexpression. The number above the left bar graph was the 2^-ΔΔCT^ value ratio, and the number above the right bar graph was the gray value ratio. Using the average to approximate the silence or overexpression efficiency. (A) Analysis of silencing or overexpression TG2 gene efficiency. Compared with the normal group, the silencing and overexpression efficiency was about 70% and 230% at the level of transcription, 15% and 210% at the level of translation. (B) Analysis of silencing or overexpression TRPC1 gene efficiency. Compared with the normal group, the silencing and overexpression efficiency was about 67% and 263% at the level of transcription, 14% and 197% at the level of translation. (C) Analysis of silencing or overexpression TRPC6 gene efficiency. Compared with the normal group, the silencing and overexpression efficiency was about 71% and 233% at the level of transcription, 17% and 211% at the level of translation.
